# “Never Trust to General Impressions, My Boy, but Concentrate Yourself upon Details”: An Unusual and Challenging Presentation of Pheochromocytoma

**DOI:** 10.3390/jcdd8060071

**Published:** 2021-06-15

**Authors:** Umberto Barbero, Mario Matta, Mirko Parasiliti Caprino, Francesca Maletta, Giuseppe Giraudo, Simone Frea, Michele De Benedictis, Mauro Maccario

**Affiliations:** 1 Division of Cardiology, “Santissima Annunziata” Hospital, 12038 Savigliano, Italy; michele.debenedictis@aslcn1.it; 2 Division of Cardiology, Hospital “Sant’Andrea” of Vercelli, 13100 Vercelli, Italy; m.matta26@gmail.com; 3 Division of Endocrinology, Diabetology and Metabolism, Department of Medical Sciences, ‘Città della Salute e della Scienza’ Hospital, University of Turin, 10126 Turin, Italy; mirko.parasiliticaprino@unito.it (M.P.C.); mauro.maccario@unito.it (M.M.); 4 Division of Pathology, Department of Laboratory Medicine, ‘Città della Salute e della Scienza’ Hospital, University of Turin, 10126 Turin, Italy; francesca.maletta@unito.it; 5 Division of General Surgery 1, Department of Surgery, ‘Città della Salute e della Scienza’ Hospital, University of Turin, 10126 Turin, Italy; giuseppe.giraudo@unito.it; 6 Division of Cardiology, Department of Clinical Sciences, University of Turin, 10126 Turin, Italy; frea.simone@gmail.com

**Keywords:** pheochromocytoma, acute cardiogenic shock, acute myocarditis

## Abstract

We present the case of a 45-year-old woman admitted to our unit with acute heart failure and cardiogenic shock, requiring an intra-aortic balloon pump insertion and inotropes and vasopressors infusion. Despite such treatment, the patient developed multi organ failure and intravascular disseminated coagulation with haemolysis. The initial diagnosis of acute myocarditis was subsequently denied by the finding of bilateral adrenal masses by MRI scan, and urine and plasma metanephrines measurements confirmed a pheochromocytoma (PCC). Genetic analysis revealed a mutation in the neurofibromatosis type 1 (NF1) gene, and an accurate physical examination drew attention to small cafè-au-lait spots, usually associated with this syndrome. PCC diagnosis should be promptly considered in patients presenting with unexplained acute heart failure and cardiogenic shock of unknown origin, considering its life-threatening complications and the good prognosis after radical surgery.

## 1. Introduction

Acute cardiogenic shock is a challenging clinical presentation of several acute cardiac conditions.

Extensive physical examination is mandatory in each patient with this kind of illness, in order not to focus exclusively on cardiogenic shock features, but also on more subtle signs that can hint at other differential diagnoses, such as skin abnormalities.

## 2. Case Report

**Figure 1:**
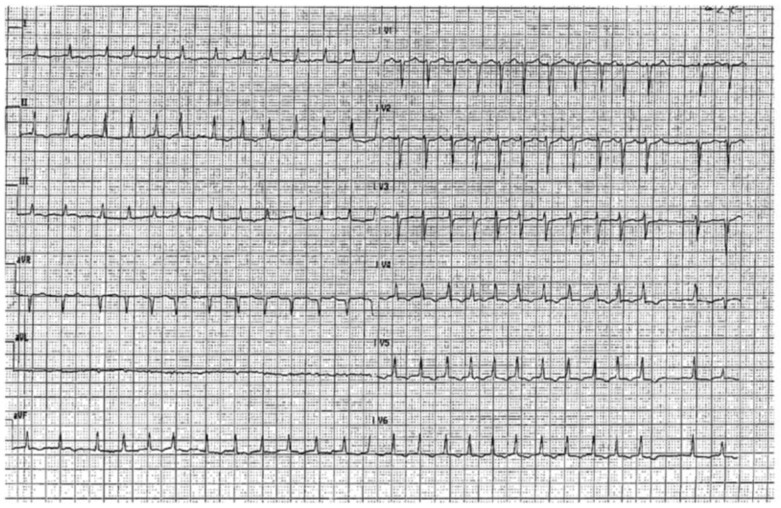
EKG at admission.

A 45-year-old woman was admitted to our emergency care unit presenting rapidly worsening dyspnoea, orthopnoea and fatigue. No prior cardiologic disease or chronic medical treatment were reported. At first medical contact, she presented severe hypotension (systolic blood pressure 80 mmHg) and oliguria. ECG showed rapid atrial fibrillation (150 bpm) of unknown onset time, with non-specific repolarisation alterations (Figure 1). Echocardiography showed normal left ventricular dimensions with severely depressed ejection fraction (LVEF), about 20%, characterised by diffuse hypokinesis. Laboratory findings were remarkable for a slight increase in myocardial necrosis markers, without a significative time-activity slope, leucocytosis, signs of acute renal and hepatic failure and acidosis with lactate increase.

The patient was immediately referred to the cath lab and underwent coronary angiography, which excluded coronary disease. Suspecting acute myocarditis, an intra-aortic balloon pump (IABP) was positioned on top of dopamine, with mild improvement of hemodynamic conditions, mean blood pressure and urine output. Acute renal failure was managed with renal replacement therapy. During the first day of hospitalisation, the patient developed a coagulopathy characterised by spontaneous INR increase, fibrinogen and anti-thrombin III reduction, severe platelet reduction and bilirubin increase, consistent with intravascular disseminated coagulation. Furthermore, several episodes of massive melena occurred, requiring red blood cells transfusion. The coagulopathy was treated with plasma and platelet transfusion for 10 days, with progressive normalisation. Empiric antibiotic treatment with piperacillin and tazobactam was set. Meanwhile, the hemodynamic state progressively improved: IABP was removed on the third day, LVEF progressively increased to 50% about 10 days after the acute presentation. Atrial fibrillation was managed with digoxin and anticoagulation with heparin. The consultant geneticist suggested a clinical diagnosis of type 1 neurofibromatosis, based on the evidence, at physical examination, of café-au-lait spots, small skin fibromas and axillary and inguinal freckling.

**Figure 2:**
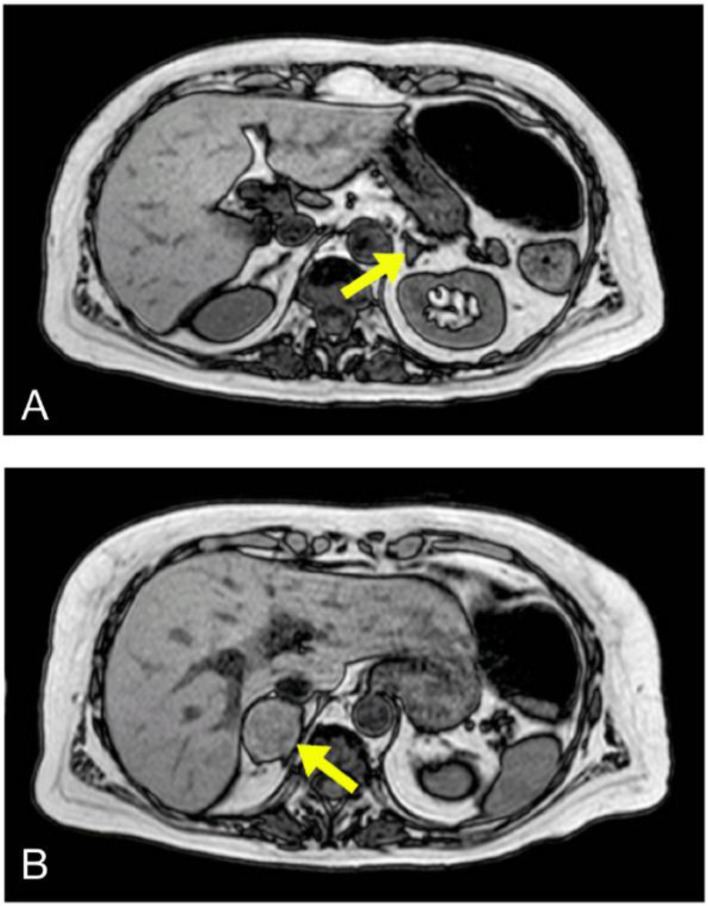
In panel (**A**) the MRI image of left PCC (6 mm) and in panel (**B**) the MRI image of right PCC (40 mm).

Despite normalisation of coagulation, renal function and LVEF, high levels of direct bilirubin persisted; therefore, an abdominal echo scan was performed, highlighting dilated biliary ducts, without hepatic masses or alterations, normal kidney size and features and a right adrenal mass. Consequently, the patient underwent an abdominal magnetic resonance imaging (MRI), which showed an ampulloma (20 mm) and both left (6 mm) and right (40 mm) adrenal glands masses (Figure 2). Following such findings, urine and plasma-fractionated free metanephrines levels were measured and found elevated (P-normetanephrines: 1518 and 2480 pmol/L, P-metanephrines: 3313 and 4222 pmol/L, U24h-nometanephrines: 437 and 509 mcg/die, U24h-metanephrines: 1386 and 1830 mcg/die). Moreover, high chromogranin A (>1100 ng/mL) and neuron-specific enolase (19.6 ng/mL) levels were consistent with the diagnosis of pheochromocyroma (PCC). The presence of metastasis was excluded with total body CT scan and with 123I-MIBG and 111In-Pentetreotide scintigraphies. Both imaging methods surprisingly showed more intense tracer uptake of left PCC mass, without visualising the ampulloma.

All tumours were surgically removed without complications. Pathologists confirmed the diagnosis of bilateral PCC (right adrenal gland: PCC of 40 mm, Ki67 2%; left adrenal gland: PCC of 5 mm with vascular invasion, profound nuclear pleomorphism, mitosis: 6 × 10 HPF, Ki67: 9%) and of ampullary neuroendocrine tumour (NET of 23 mm, G2 with muscular invasion—pT2 N0 M0). At discharge, therapy was as follows: propranolol, hydrocortisone, fludrocortisone, furosemide, potassium supplement and trans-dermic nitrates.

Results from genetic analysis confirmed the mutation in NF-1 gene. At one-year follow-up visit, the patient presented good general health conditions and has no biochemical or radiological evidence of disease recurrence.

## 3. Discussion

This is a rare case of bilateral PCC in NF-1 presenting with acute heart failure and cardiogenic shock. At first, on account of clinical presentation, all diagnostic and therapeutic approaches were addressed by the hypothesis of acute myocarditis. As a matter of fact, the patient required hemodynamic support, while multi-organ failure, coagulopathy and melena were considered as consequences of the cardiogenic shock.

The diagnosis of PCC, and therefore the appropriate surgical treatment, was only assessed after a few weeks, due to the persistence of impaired hepatic function along with newly diagnosed hypertension. Arguably, an earlier diagnosis would have headed towards the proper treatment more rapidly, saving time and tests.

Several previous case reports described an acute presentation of PCC as a tako-tsubo-like cardiomyopathy [1,2], sometimes featured by acute heart failure and cardiogenic shock. However, unlike our patient, in these reports patients presented with chest pain before signs and symptoms of heart failure. More considerably, our patient did not present any typical tako-tsubo cardiomyopathy ECG and echocardiographic features. In fact, the typical or inverted tako-tsubo-mimicking presentation has also been reported among patients with PCC in single case reports [3,4,5,6], leading to acknowledge PCC as a possible cause of tako-tsubo-like cardiomyopathy, since its massive catecholamine release results in abnormal adrenergic stress [7,8].

In our case, PCC featured as an acute fulminant myocarditis [9], and high inflammatory markers were consistent with this hypothesis. The correct diagnostic work-up was guided by the persistence of elevated cholestasis markers, suggesting an abdominal disease. Indeed, PCC was identified by diagnostic exams performed to characterise the biliary duct mass. 

More noticeably, we discuss a very rare case, as bilateral PCC occurs only in 0.6% of patients with NF-1 [10] and ampullary neuroendocrine tumour can be included in the clinical manifestations of the syndrome.

## 4. Conclusions

This case suggests that PCC should be promptly considered in critical care of patients presenting with cardiogenic shock, no coronary artery disease and clinical features of alleged myocarditis or tako-tsubo syndrome. Acute cardiogenic shock is a challenging clinical presentation of different acute cardiac conditions. An extensive physical examination is mandatory in each case not to miss the clues for differential diagnosis between common causes, such as myocarditis and tako-tsubo syndrome, and other infrequent conditions, such as pheochromocytoma.

As Sherlock Holmes stated in “The Adventure of the Blue Carbuncle”, usually in medicine we should mistrust the first general impression and concentrate on details: in this case, an accurate physical examination at admission could have revealed *cafè-au-lait* spots, freckles or cutaneous neurofibromas, potentially directing to appropriate urine and plasma analyses, and possibly to abdominal imaging.
